# First record of water Mite (*Arrenurus* spp.) ectoparasitism on dragonflies (Odonata: Anisoptera) in Bangladesh

**DOI:** 10.1016/j.ijppaw.2026.101303

**Published:** 2026-10-13

**Authors:** Khondoker Md Zulfiker Rahman, Farjana Akter, Md Shohag Mia

**Affiliations:** Laboratory of Entomology, Department of Zoology, Jahangirnagar University, Dhaka, 1342, Bangladesh

**Keywords:** *Arrenurus*, Dragonfly, Water mite, Infestation, Bangladesh

## Abstract

Water mites, as ectoparasites, are known to significantly impact the flight behavior, fitness, and reproductive success of their aquatic insect hosts. Although water mite infestations on dragonflies have been documented globally, the ectoparasitic relationship between dragonflies and water mites has remained completely unexplored in Bangladesh. Therefore, for the first time in Bangladesh, this study investigated the prevalence, intensity, host selection, sex bias, and site specificity of larval water mite infestation in dragonflies in a semi-urban lentic ecosystem on the Jahangirnagar University campus, Bangladesh, from June 2023 to April 2024. During the study period, 2627 dragonfly individuals from 20 species were checked for the presence of water mite larvae; 318 individuals from 6 species were found infested, yielding an infestation prevalence of 12.11%. All the larvae were identified as a single genus, *Arrenurus* sp. Among the infested dragonflies, the highest of 35.60% prevalence was exhibited by *C. servilia*, and the highest mean parasitic intensity was recorded in *A. panorpoides* (13.75 mites/host). Most of the mite larvae (83.51%) were attached ventrally to the thoracic segments, whereas the ventral surface of the abdomen and wings harboured 16.44% and 0.05% mite larvae, respectively. Besides, a female-biased infestation trend was observed, with overall parasitic intensity of 13.18 and 11.62 in female and male dragonflies, respectively. The findings of this study indicate that dragonflies in the sampling area are actively parasitized by *Arrenurus* water mite larvae, thereby providing nutrition and dispersal for these ectoparasites in freshwater ecosystems of Bangladesh. These preliminary results reveal a previously unexplored expansion in the geographic range of ectoparasitic interactions between *Arrenurus* water mite larvae and Odonata and warrant the need for additional research into the potential long-term fitness implications of these ectoparasites for tropical dragonfly populations.

## Highlights

• First study reporting *Arrenurus* mite larvae in dragonflies in Bangladesh.
• Overall prevalence of infestation was 12.11%; highest in *C. servilia* at 35.60%.
• Mites were localized on the ventral thorax and abdomen and rarely on the wings.
• A female host bias in parasitic load was observed.
• Further research on *Arrenurus* mite larvae on dragonflies is needed in the tropics.

## Graphical abstract

**Figure:**
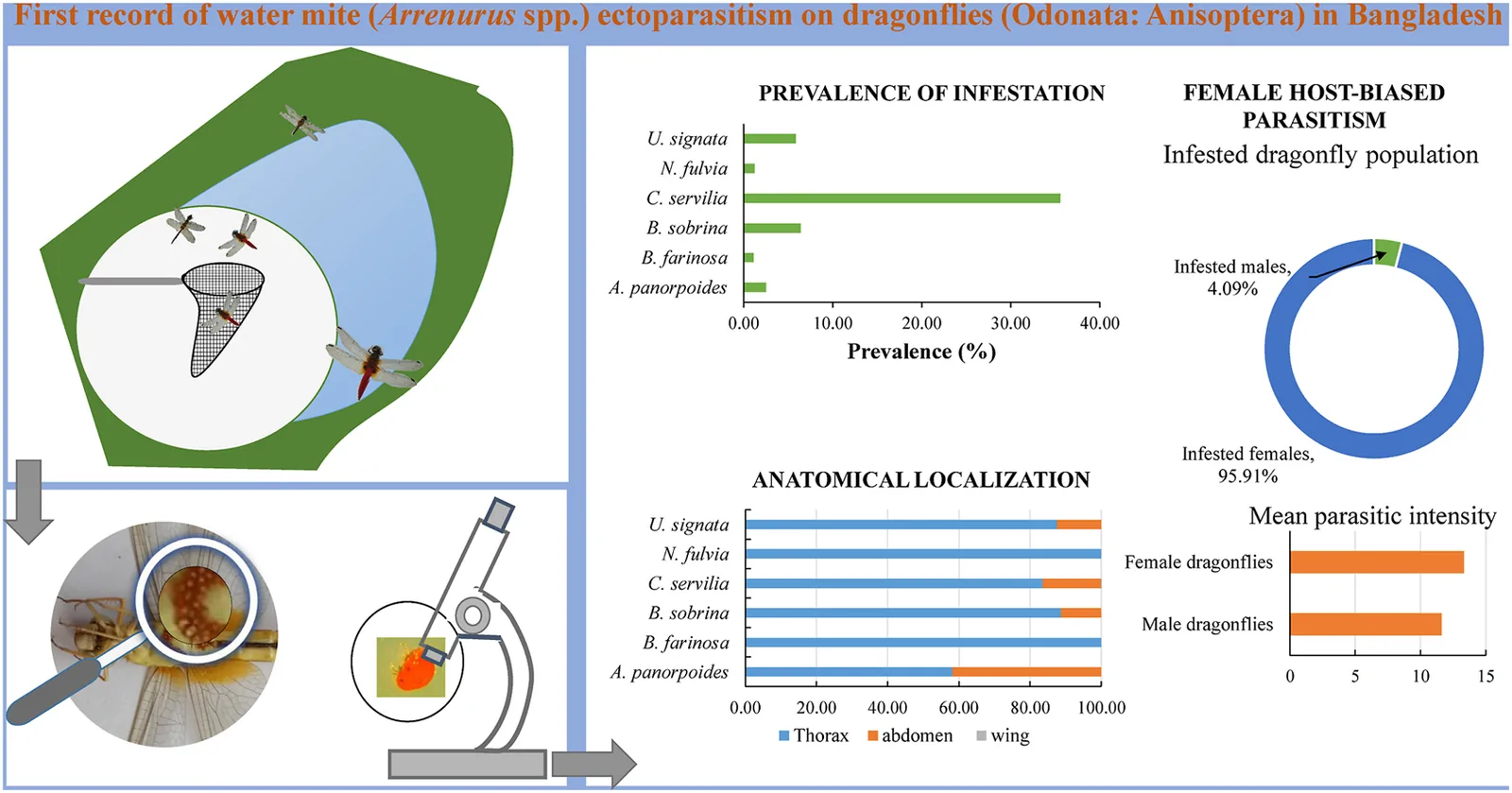

## 1 Introduction

Dragonflies (Odonata: Anisoptera) serve as one of the basic structural and functional elements of freshwater and adjacent terrestrial ecosystems. With the immature being stages aquatic and the adults being terrestrial, they can serve as vital ecological bridges between aquatic and terrestrial ecosystems, thereby facilitating cross-ecosystem transfer of nutrients, biomass, and energy. Throughout the life cycle, dragonflies act as dominant generalist predators; i.e., the aquatic nymphal stage holds the top trophic position among macroinvertebrates, thereby regulating the population densities of mayfly and stonefly nymphs, caddisfly larvae, midge larvae, mosquito larvae, and small predatory invertebrates. After emerging as adults, dragonflies continue this top-down approach to their regulatory role by preying on flying insects, including pests of agricultural importance and adult disease-vector mosquitoes. Moreover, their presence and diversity serve as bioindicators of environmental health, climate change, and water quality ( Zawal and Jaskuła, 2008).

Among aquatic macroinvertebrates, odonates are highly susceptible to ectoparasitism by water mite larvae, particularly those of the genus *Arrenurus* (Smith and Oliver, 1986). Over 55 species of aquatic mites in the genus *Arrenuru*s act as ectoparasites for Odonata (Corbet, 1999). During the emergence of the adult odonates from the aquatic nymphal stage, the mite larvae attach to their body by means of their chelicerae and feed on the host's haemolymphs, thus gaining nutrients needed to transform into a deutonymph, which cannot live longer in the terrestrial environment. When adult odonates return to the water body for mating and oviposition, the deutonymphs drop into the water, enter a predatory phase, and eventually develop into adults (Di Sabatino et al., 2000; Zawal, 2006).

The behavior, structure, physical condition, and overall health of a host are affected by parasites. As a result, they can alter interactions related to competition and predation among hosts and other species, thereby affecting During periods of extensive breeding, these parasitic larvae adversely affect various physiological aspects of odonates, including their aerial performance, longevity, and fertility, thereby decreasing population levels community dynamics and biodiversity patterns (Åbro, 1982; Forbes and Baker, 1991; Reinhardt, 1996).

Disentangling the complex ectoparasitic relationship of aquatic mites with their host dragonflies is crucial to the successful conservation of dragonfly populations within an ecosystem. By doing so, researchers and conservationists can assess ecosystem status, design management strategies that support dragonfly populations, predict population dynamics, and prioritize conservation efforts. However, despite the global attention aquatic mite-dragonfly ectoparasitic interactions have received (Andrew et al., 2012; Janra and Herwina, 2021; Zawal, 2004), this area has been totally overlooked in Bangladesh, where research has primarily focused on assessment of dragonfly diversity (Bashar et al., 2014), their role as bioindicators (Haque et al., 2025) and biocontrol agents of mosquitoes (Rahman et al., 2022). Hence, the present study was undertaken to assess the prevalence, intensity, host selection, and sex bias of larval water-mite infestation in dragonflies within a semi-urban lentic ecosystem in Bangladesh.

## 2 Materials and methods

### 2.1 Study area

The present study was conducted at the Jahangirnagar University campus in Bangladesh (Fig. 1). The campus is located approximately 32 km northwest of Dhaka, the capital of Bangladesh, and hosts a unique semi-urban, semi-natural ecosystem spanning approximately 697.56 acres. The campus landscape belongs to the Madhupur Tract of the Pleistocene period and features gently undulating topography and reddish-brown iron-enriched soil, ultimately creating a suitable environment that supports the local rich biodiversity. The climate of the area can be divided into three distinct seasons: a monsoon with heavy rain (June-October), a hot summer (March-May), and a dry, cooler winter (November-February). There is an extensive network of freshwater lentic water bodies, including more than 20 lakes ranging from small to large, as well as many human-made ponds and shallow depressions known as dobas. The water levels in these water bodies exhibit dynamic seasonal volume variations due to monsoonal flush and runoff from residential buildings. The littoral surface of water bodies hosts dense aquatic macrophytes, particularly lotus, water lilies, water hyacinth, and other floating vegetation mats. These unmaintained water bodies created suitable habitats for aquatic macroinvertebrates, including odonate nymphs and water mite larvae.

**Fig. 1:**
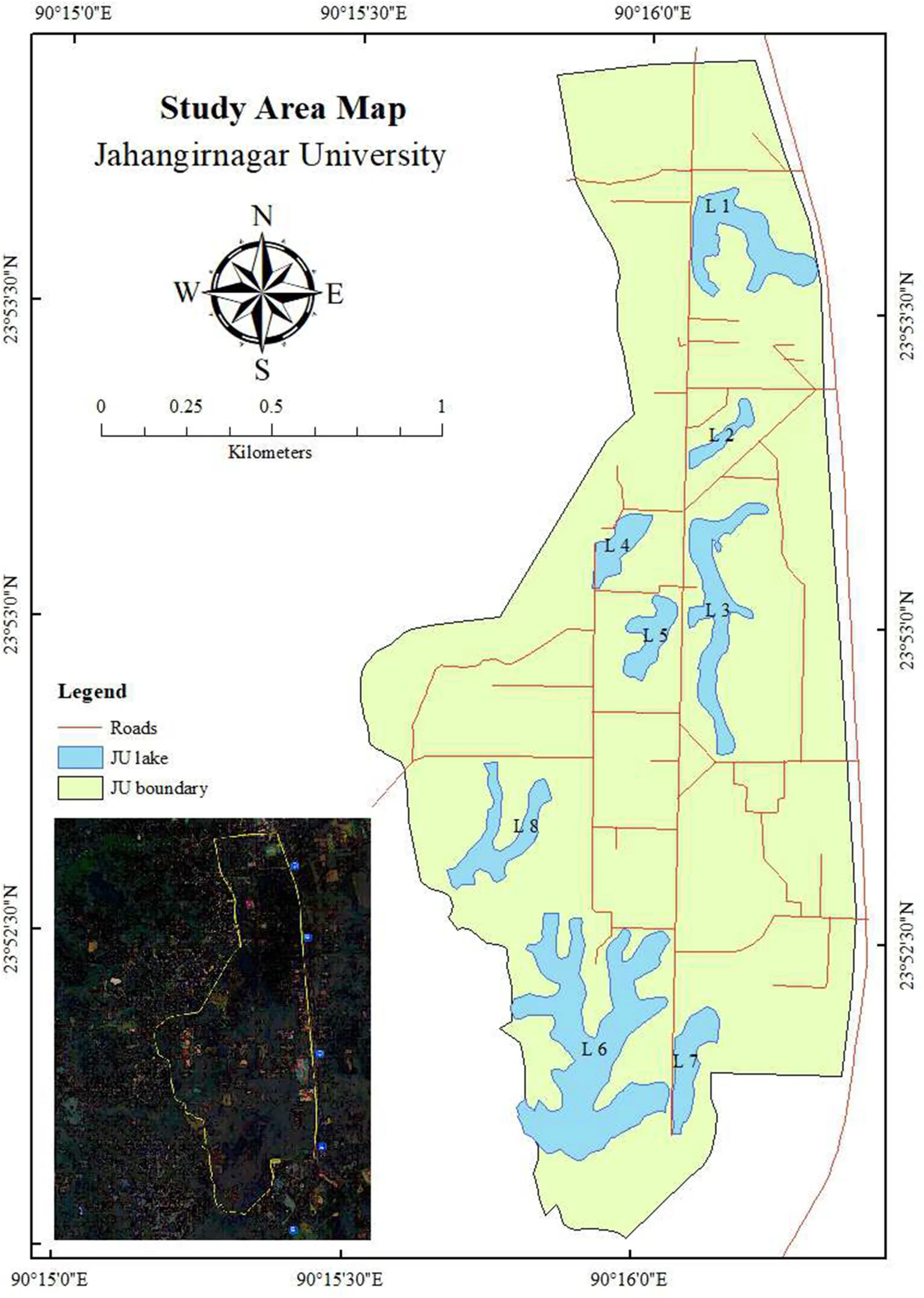
Map of the study area. L1-L8 with blue shades represent major lakes at the study area.

Additionally, the campus hosts more than 900 species of vascular plants. Thus, the campus serves as a hub for a regional biodiversity hotspot, hosting a variety of arthropods, amphibians, reptiles, birds, and small mammals. The presence of various dragonfly species on the university campus offers an opportunity to investigate habitat preferences and to identify susceptible host species for water mites.

### 2.2 Collection and identification of dragonflies

Adult dragonflies were collected twice a month from June 2023 to April 2024 at Jahangirnagar University campus. Sampling was carried out at different microhabitats, i.e., riparian zones and water surfaces of the 8 major lakes (Fig. 1), road sides, grasslands, and dense tree canopies during the flight activity period of dragonflies between 09:00 a.m. and 03:00 p.m. using sweep nets. The dragonflies were handled with caution to avoid any mechanical damage. The dragonflies were identified to species level using suitable taxonomic keys (Fraser, 1934, 1936; Orr, 2005).

### 2.3 Collection and preservation of mites

Water mite larvae were separated from the infested dragonfly using a soft brush and transferred to a separate Petri dish. The larvae were then counted and imaged under a stereomicroscope. Identification of water mite larvae was carried out following established taxonomic keys (Prasad and Cook, 1972; Zawal, 2008). Few dragonfly specimens were preserved in 70% alcohol, and some were also dried in a drying oven for 24-48 h, depending on their size. After drying, the specimens were carefully preserved in wooden boxes, labeled with the date and location for future reference and study. Other dragonflies were released after data collection.

## 3 Results

### 3.1 Host prevalence and population dynamics

A total of 2627 individuals of dragonflies belonging to twenty (20) species under two families were examined during the study period. Nineteen (19) of these species belonged to the Libellulidae family, while only one species was classified under the Gomphidae family (Table 1). Among the dragonfly specimens examined for the presence of water mites, 318 instances of dragonflies belonging to six species were parasitized by water mite larvae belonging to the genus *Arrenurus* Dugès 1834, resulting in an overall host prevalence of 12.11%. All the mite larvae-infested dragonflies belonged to the family Libellulidae and included the following species: *Acisoma panorpoides* (4), *Brachydiplax sobrina* (9), *B.farinosa* (1), *Crocothemis servilia* (288), *Neurothemis fulvia* (1), and *Urothems signata* (15). Parasitic prevalence varied drastically among the susceptible hosts. The prevalence of infestation was highest in *Crocothemis servilia* (35.60%), followed by *B. sobrina* (6.38%), *U*. *signata* (5.84%), *A. panorpoides* (5.06%), *N. fulvia* (1.25%), and *B. farinosa* (1.15%). The remaining 14 species—including *Pantala flavescens* (n = 189), *Rhyothemis variegata* (n = 438), *Orthetrum sabina* (n = 141) and *Brachythemis chalybea* (n = 126) showed zero infestation (Table 1, Fig. 2).

**Table 1:** Prevalence of *Arrenurus* aquatic mites on dragonflies at JU Campus.

Dragofly species	No. dragonfly sampled	No. dragonfly infested	Prevalence of infestation
*Acisoma panorpoides*	79	4	5.06
*Brachythemis contaminata*	14	0	0.00
*Bradinopyga geminata*	6	0	0.00
*Brachydiplax chalybea*	126	0	0.00
*B. farinosa*	87	1	1.15
*B. sobrina*	141	9	6.38
*Crocothemis servilia*	809	288	35.60
*Diplacodes trivialis*	9	0	0.00
*Ictinogomphus rapax*	38	0	0.00
*Neurothemis fulvia*	80	1	1.25
*N. tulia*	83	0	0.00
*Orthetrum pruinosum*	68	0	0.00
*O. sabina*	57	0	0.00
*Potamarcha congener*	15	0	0.00
*Pantala. flavescens*	189	0	0.00
*Rhodothemis rufa*	46	0	0.00
*Rhyothemis variegata*	438	0	0.00
*Tramea limbata*	1	0	0.00
*Tholymis tillarga*	84	0	0.00
*Urothemis signata*	257	15	5.84
Total/average	2627	318	12.11

**Fig. 2:**
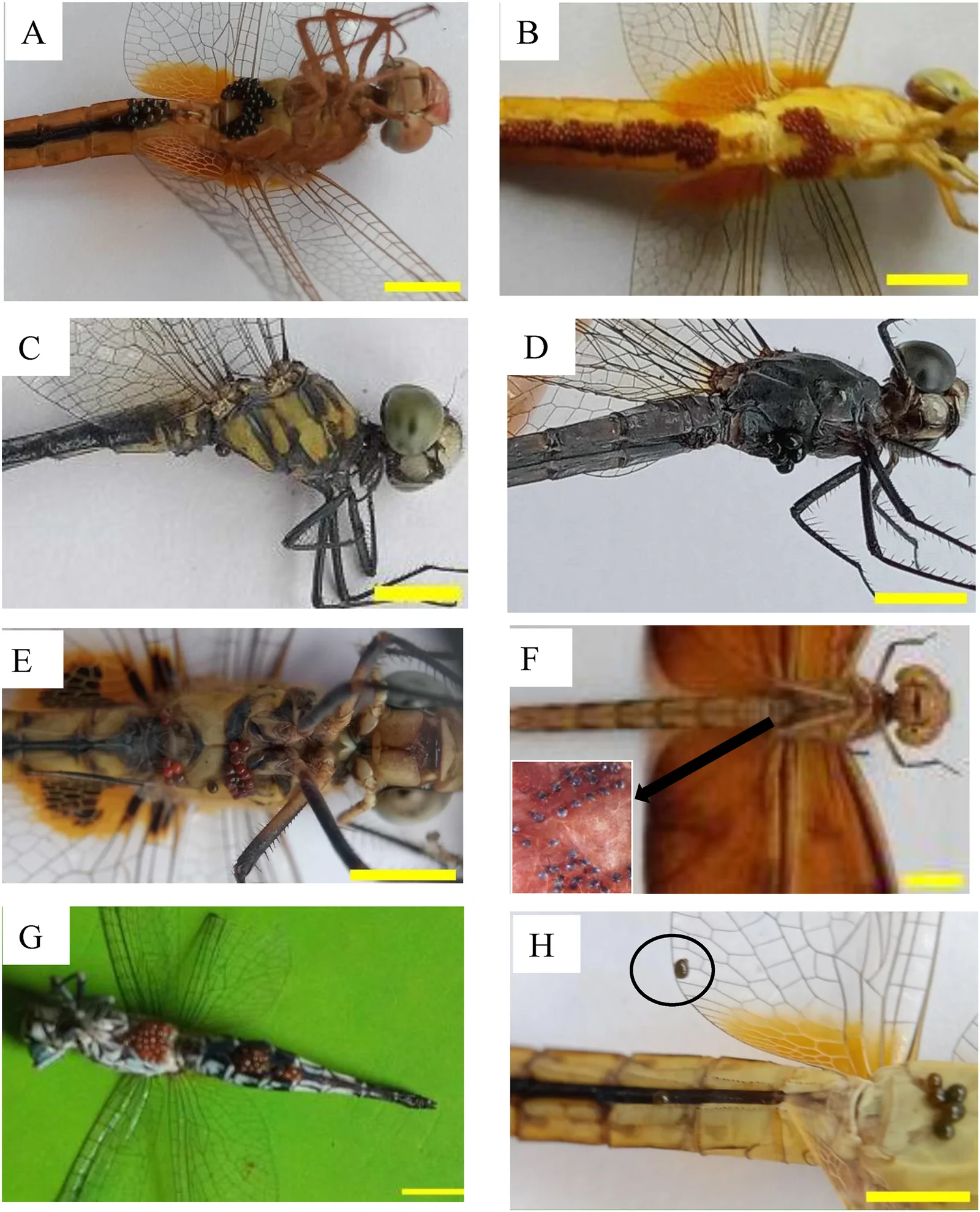
Ventral and lateral view of six dragonfly species (Panel A-H) infested by *Arrenurus* water mites. (A) *C. servilia* male (ventral view), (B) *C. servilia* female (ventral view), (C*) B. sobrina* female (lateral view), (D) *B. farinosa* male (lateral view), (E) *U. signata* female (ventral view), (F) *N. fulvia* female, zoomed-in thorax inset (ventral view), (G) *A. panorpoides* male (ventral view), (H) *C. servilia* female infested on wings (black circle). Scale bar = 5 mm (added using imageJ).

### 3.2 Anatomical distribution on host bodies

Localization of mite larvae within the host body indicated a strong preference for specific regions, with the thorax as the primary attachment site. In the case of severe infestations, the mites were attached to the ventral surface of both the thorax and abdomen. Sometimes they heavily infested the thorax or both the thorax and the abdomen. In a few cases, infested mites were found in the wing regions (Fig. 2).

Out of 4172 localized mites, 3484 mites accounted for 83.51% of the total mites were attached to the thoracic segments, whereas the abdominal segments harbored 686 mites (16.44%), and the wings had only 2 mites (0.05%). Within the thoracic region, mesothorax and metathorax harbored a vast majority of the mites, with 2096 and 188 mites, respectively, while the prothorax was completely free of mites across all species. In the abdominal region, mites preferred to attach to the anterior segments, particularly on segment 2 (n = 203) and segment 3 (n = 130). The number of mites gradually declined toward the posterior segments and entirely disappeared on segments 9 and 10 (Table 2).

**Table 2:** Anatomical distribution of *Arrenurus* aquatic mites on dragonflies at JU Campus.

Host species	Thoracic segments					Abdominal segments												Wings				Total
	Pro-thorax	Meso-thorax	Meta-thorax	Sub-total	% Total	1	2	3	4	5	6	7	8	9	10	Sub-total	% Total	1	2	Sub-total	% Total	
*A. panorpoides*	0	7	25	32	58.18	0	17	6	0	0	0	0	0	0	0	23	41.82	0	0	0	0	55
*B. farinosa*	0	0	6	6	100.00	0	0	0	0	0	0	0	0	0	0	0	0	0	0	0	0	6
*B. sobrina*	0	10	37	47	88.68	4	2	0	0	0	0	0	0	0	0	6	11.32	0	0	0	0	53
*C. servilia*	0	2000	1286	3286	83.53	83	183	122	74	66	45	37	36	0	0	646	16.42	0	2	2	0.051	3934
*N. fulvia*	0	32	3	35	100.00	0	0	0	0	0	0	0	0	0	0	0	0	0	0	0	0	35
*U. signata*	0	37	41	78	87.64	1	1	2	6	1	0	0	0	0	0	11	12.36	0	0	0	0	89

Total	0	2086	1398	3484		88	203	130	80	67	45	37	36	0	0	686			2	2		4172

The preference for microhabitat was also observed in individual dragonfly species examined. Out of the 3934 mites harbored by *C. servilia*, 3286 mites (83.54%) were attached on the thorax compared to 646 (16.42%) on the abdomen and only 2 (0.05%) on the wings. This thoracic preference was mirrored in *A. panorpoides* (32 thoracic, 23 abdominal), *B. sobrina* (47 thoracic, 6 abdominal), and *U. signata* (78 thoracic, 11 abdominal). In *N. fulvia* and *B. farinosa*, mites were found exclusively on the thorax, leaving the abdomen and wings entirely uninfected (Table 2, Fig. 2).

### 3.3 Sex-biased parasitism

In the present study, 4172 *Arrenurus mite* larvae were observed across 318 infested dragonfly hosts, yielding an overall mean parasitic intensity of 13.12. Among the hosts infested with multiple individuals of mites (n > 1), the mean parasitic intensity was highest in *A. panorpoides* (13.75 mites/host, n = 4), followed by *C. servilia* (13.66 mites/host, n = 288), *U. signata* (5.93 mites/host, n = 15), and *B. sobrina* (5.89 mites/host, n = 9). Because single-host infestation occurred in *N. fulvia* (35 mites infesting 1 female host) and *B. farinosa* (6 mites infesting 1 male host), the mean parasitic intensity for these two species was not calculated to avoid skewing interspecific comparisons (Table 3).

**Table 3:** Number of *Arrenurus* aquatic mites on male and female dragonflies at JU Campus.

*Host species*	No.of infested hosts			No. of mites			aMean parasitic intensity			
	Male	Female	Total	Male	Female	Total	Host species	Male hosts	Female hosts	Overall
*A. panorpoides*	4	0	4	55	0	55	13.75	11.62	13.18	13.12
*B. farinosa*	1	0	1	6	0	6	b			
*B. sobrina*	0	9	9	0	53	53	5.89			
*C. servilia*	8	280	288	90	3844	3934	13.66			
*N. fulvia*	0	1	1	0	35	35	b			
*U. signata*	0	15	15	0	89	89	5.93			
*Total*	13	305	318	151	4021	4172				

a Total number of mites divided by number of infested dragonfly host.

b Due to single-host infestation, the mean parasitic intensity was not calculated in *N. fulvia* and *B. farinosa*.

The results of this study showed that the mites preferred to infest the female dragonfly hosts. Mites were found in 305 female dragonfly hosts out of a total of 318 sampled dragonfly population, accounting for 95.91% of the prevalence rate. These individuals harbored 4021 mites, accounting for 96.38% of the total mites collected. On the contrary, male dragonflies comprised only 13 individuals, which accounted for 4.09% of infested individuals and harbored 151 mites, which accounted for 3.62% of the total mite population. The mean parasitic intensity in female hosts (13.18 mites/host) was higher than in males (11.62 per host) (Table 3).

Pronounced sex-skewed parasitism was also evident when individual species were considered. In *C. servilia*, out of 288 infested individuals, 280 (97.22%) were females, whereas only 8 (2.78%) were │males. More importantly, this is the only species in our study in which parasitism was recorded in both males and females. Interestingly, mite infestation was entirely restricted to female hosts in *B. sobrina* (n = 9), *N. fulvia* (n = 1), and *U. signata* (n = 15). Conversely, *A. panorpoides,* and *B. farinosa* showed exclusively male-biased infestation patterns, with mean parasitic intensities of 13.75 (n = 4) in the former and 6 mites (n = 1) in the latter (Table 3, Fig. 2).

## 4 Discussion

### 4.1 Prevalence and population dynamics of hosts

Infestation of *Arrenurus* mite larvae in 6 out of the 20 sampled dragonfly species indicates a high degree of host prevalence. These results are in line with a previous study in Central India, where *Arrenurus* water mites showed strong preferences for certain dragonfly species, while being completely absent in others sharing the same habitat (Andrew et al., 2012). The highest susceptibility recorded in *C. servilia* indicates that this species serves as the primary reservoir host for water mite larvae in the area. These findings are consistent with a previous study (Andrew et al., 2015), which also reported the highest prevalence of infestation in *C. servilia*. Differential host-prevalence rates among the dragonfly species examined might be due to behavioral and ecological overlaps of mites during host emergence. Larvae of *Arrenurus* water mites attach temporarily to final-instar odonate nymphs during their emergence to adults in water and transition to active ectoparasitism while the host molts into a flying adult (Abro, 1979; Smith et al., 2001). Therefore, the greater prevalence of these mite larvae in *C. servilia* could be attributed to their nymphal microhabitat significantly overlapping with the benthic or macrophyte zones preferred by mite larvae, or to their emergence strategy, which involves soft-bodied adults that are more susceptible to colonization for longer periods. Conversely, complete resistance in some abundant dragonfly species in the study area, such as *R. variegata* (n = 438), *P. flavescens* (n = 189), and *B. chalybea,* to *Arrenurus* mite larvae might be due to temporal, spatial, or immunological disparities that keep them isolated from parasitic pressure.

### 4.2 Anatomical distribution on host bodies

All the mites observed in this study were found attached to the underside of both the thorax and the abdominal region, except for two instances attached to a hind wing of *C. servilia*. Our findings are consistent with several other studies (Kulijer et al., 2012; Zawal, 2004), which reported that the ventral side of odonates, the thorax and abdomen, in particular is the major location where water mites are known to attach. Another study in Monetnegro showing that the anisopteran dragonfly, *Sympetrum meridionale*, carries water mite larvae on its wings, with more on the hindwings than on the forewings, also supports our study Within the body of dragonflies, the ventral portions of the thorax and abdomen are softer due to a lack of sclerotization (Tillyard, 1917). Consequently, parasites can easily attach themselves to these soft regions using their sharp mouthparts, adhesive parts, or claws, thereby, feeding on the body fluids. This preference arises from the lower parasite mortality in this area, as odonate legs protect against mechanical injury (Zawal and Buczyński, 2013). In addition, the thorax emerges first from the exuvium (Bhende et al., 2024) and may serves as the primary site of attachment of mites. When the thorax is fully occupied, the first abdominal segments are occupied, then the others. However, in our study, we observed that water mites localized to the ventral abdomen were restricted to the first 8 abdominal segments. Interestingly, the distal abdominal segments, which are most beneficial for the mite in providing the closest contact with water during oviposition, have not been used by mites in our study, probably because those segments are more vulnerable to mechanical damage, which might cause mortality of larval mites.

### 4.3 Sex-biased parasitism

The results of this study showed a female-host bias in both host prevalence (95.91%) and overall mite population distribution (96.38%). The higher mean parasitic intensity in female *C. servilia* than in their male counterparts closely resemble findings from Central India, which reported that 71% and 85.7% of all mite-infested dragonflies examined during the post-monsoon and summer months, respectively, were females (Andrew et al., 2012). The underlying reason for the preference for females might be the reproductive traits of the host individuals. The hosts may provide a more nutrient-rich environment for mites through vitellogenesis during egg maturation. As the mites feed on hemolymph, this may facilitate faster development in female hosts. From an evolutionary perspective, female-biased parasitism provides a significant adaptive advantage for water mites. Mite larvae entirely depend on their host to return to an aquatic environment. Visiting the water surface for oviposition is a common behavior in female dragonflies. Thus, they infest female hosts, ensuring that mites will return to the aquatic habitat and begin their life cycle as predatory deutonymphs. However, the exclusive male-skewed infestation in *A. panorpoides* and *B. farinosa* might be attributed to certain territorial behaviors of these host species that may increase their exposure to specific niches, particularly near water margins. Besides, certain specific localized microclimates, such as humidity, temperature, or resource availability, may create a favorable environment for the activity and survival of water mite larvae. Such variations in those microclimates could create specific habitats favorable for a flowerish male population, thus ensuring a richer male representation in those areas than in more stable habitats.

## 5 Conclusions

This study provides the first documentation of the ectoparasitic relationship of larval water mites with adult dragonflies in Bangladesh. The results of this study reveal that these ectoparasitic water mites exhibit a strong pattern of host preference, anatomical site specificity, and sex bias. The preliminary data obtained from this research successfully fill a long-standing geographical gap in tropical water mite-odonate interactions within South Asia. Since dragonflies are regarded as bioindicators and among the top predators in both aquatic and terrestrial ecosystems, identifying the factors that affect their fitness is crucial for their conservation at the local scale. Mite infestation, although less studied in dragonflies, poses a potential threat to their populations. Hence, this novel work opens a new avenue for future parasitological and ecological inquiries, and future investigations should focus on spatio-temporal variation and the climatic factors governing water-mite ectoparasitism in dragonflies in Bangladesh.

## CRediT authorship contribution statement

**Khondoker Md Zulfiker Rahman:** Conceptualization, Funding acquisition, Supervision, Data curation, Methodology, Project administration, Resources, Software, Validation, Writing – original draft, Writing – review & editing. **Farjana Akter:** Investigation, Formal analysis, Visualization, Writing – review & editing. **Md Shohag Mia:** Investigation, Formal analysis, Formal analysis, Visualization, Writing – review & editing.

## Data availability

The analyzed datasets during the current study are available from the corresponding author upon reasonable reasonable request.

## Declaration of competing interest

The authors declare that they have no known competing financial interests or personal relationships that could have appeared to influence the work reported in this paper.
